# A systematic review and meta-analysis comparing the effect of aquatic and land exercise on dynamic balance in older adults

**DOI:** 10.1186/s12877-020-01702-9

**Published:** 2020-08-25

**Authors:** Youngwook Kim, Michael N. Vakula, Benjamin Waller, Eadric Bressel

**Affiliations:** 1 Department of Kinesiology and Health Science, Utah State University, 7000 Old Main Hill, Logan, UT 84322-7000 USA; 2 Department of Sport Science, Reykjavík University, Reykjavík, Iceland

**Keywords:** Older adults, Seniors, Aquatic exercise, Aquatic therapy, Balance, Dynamic balance, Falls, Fall prevention

## Abstract

**Background:**

Balance impairments are the leading causes of falls in older adults. Aquatic-based exercises have been broadly practiced as an alternative to land-based exercises; however, the effects on dynamic balance have not been comprehensively reviewed and compared to land exercises. Thus, the purpose of this systematic review and meta-analysis was to compare the effectiveness of aquatic exercises (AE) to land exercises (LE) on dynamic balance in older adults.

**Methods:**

Electronic databases (PubMed, MEDLINE, CINAHL, SPORTDiscus, psycINFO), from inception to November 2019, were searched. Studies met the following eligibility criteria: Randomized controlled trials, English language, older adults aged 65 years or older, a minimum of one AE and LE group, at least one assessment for dynamic balance. For the meta-analysis, the effect sizes of dynamic balance outcomes were calculated using a standardized mean difference (SMD) and a 95% confidence interval (CI).

**Results:**

A total of 11 trials met the inclusion criteria, and 10 studies were eligible for the meta-analysis. The meta-analysis presented that older adults in AE groups demonstrated comparable enhancements in dynamic steady-state balance (SMD = − 0.24; 95% CI, −.81 to .34), proactive balance (SMD = − 0.21; 95% CI, −.59 to .17), and balance test batteries (SMD = − 0.24; 95% CI, −.50 to .03) compared with those in LE groups.

**Conclusions:**

AE and LE have comparable impacts on dynamic balance in older adults aged 65 years or older. Thus, this review provides evidence that AE can be utilized as a reasonable alternative to LE to improve dynamic balance and possibly reduce the risk of falls.

## Background

In adults aged 65 years or older, approximately 29% of the population experience at least one fall per year, and the rate of falls and fall-related injuries increase with age [1]. Falls are a common cause of morbidity and mortality including both fatal and non-fatal injuries and poor quality of life [2, 3]. Falls often cause substantial medical costs. In 2015, fatal fall-related and non-fatal fall-related injuries cost an estimated $637.5 million and $31.3 billion, respectively [4]. Considering the globally increasing proportion of older adults, the medical costs related to falls may constantly increase unless cost-effective interventions are established and implemented.

Exercise interventions have been effective at improving balance and reducing fall risks in older adults [5–8]. A Cochrane systematic review by Howe et al. indicated that exercise on land is the most common form of treatment in older adults to improve balance and reduce fall risk [9]. However, land-based exercises contain a higher rate of extrinsic fall risk factors (e.g., uneven walking surface) when compared to aquatic exercises, which may, in turn, interrupt the progression of a fall prevention exercise program. This is important to note because extrinsic risk factors account for the majority of all falls [10]. These aforementioned limitations associated with the safety issues during land-based exercises are less common in aquatic-based exercise programs [11].

Aquatic exercises have been utilized as an alternative to land-based exercises for older adults that display lower physical activity levels, neuromuscular degeneration, or orthopedic disabilities that affect balance, mobility, and pain [12–14]. For this systematic review and meta-analysis, we defined the aquatic exercise as any type of exercise performed in water. The buoyant force of water and the hydrostatic pressure/density help participants slow the movement, and additional sensory cues supplied by the viscosity of water facilitate muscle recruitment timing [15]. Thus, water provides a safe, low risk, and supportive training environment, which may be advantageous for older adults to participate in exercise programs without the risk or fear of falling [16].

Previous systematic reviews have summarized empirical evidence for aquatic exercises on strength, mobility, flexibility, balance, and various health outcomes in older adults [12, 13, 17]. Observations from these reviews have indicated that aquatic exercises may improve the aforementioned outcome measures. Specifically, a recent systematic review and meta-analysis summarized statistical evidence for aquatic exercise on dynamic balance for the first time and reported that aquatic exercise significantly improved dynamic balance in older adults with knee or hip osteoarthritis [18]. However, only four studies and one outcome measure (Timed Up and Go test) were included in the meta-analysis, and the population was limited to osteoarthritic patients. Moreover, the results of aquatic exercise were compared to the controls, thus, evidence regarding the effectiveness of aquatic exercises over comparable land-based exercises in older adults is inconclusive. Due to complex environments continuously challenging older adults, various dynamic balance abilities, that can be defined as the ability to control postural stability while in motion [19], are critical in this population [20]. Accordingly, there is a need to more formally quantify the effects of AE on dynamic balance concerning fall prevention protocols. This systematic review and meta-analysis aimed to compare the effects of aquatic exercise (AE) and land exercise (LE) on dynamic balance in older adults aged 65 years or older. The PICO question was as follows: “Are aquatic exercises more effective than land-based exercises at improving dynamic balance in older adults aged 65 years or older?”

## Methods

A systematic review of the literature with meta-analysis was conducted in November 2019 to examine the effects of AE on dynamic balance in older adults. The following electronic databases were searched by one reviewer (Y.K.) on November 19th, 2019: PubMed (1965-), MEDLINE (1959-), CINAHL (1984-), SPORTDiscus (1978-), psycINFO (1958-). The databases were examined using the following combination of keywords: (aquatic therapy OR aquatic activity OR aquatic aerobics OR aquaerobics OR aquatic exercise OR aquatic physical therapy OR aquatic physiotherapy OR aquatic rehabilitation OR hydrotherapy OR pool exercise* OR pool therapy OR swimming OR swimming therapy OR water aerobics OR water-based exercise OR water exercise OR water rehabilitation OR water therapy OR water rehabilitation OR water activity, OR water sport∗) AND (aged OR older OR elderly OR senior) AND (balance OR postur*). There was no restriction on the publication year.

All articles identified in the database search were exported to Zotero 5.0.66 (http://www.zotero.org) and any duplicates were deleted. Two reviewers (Y.K. and M.V.) initially screened, included, and excluded studies based on titles and abstracts. Full text of identified articles was obtained and reviewed by the first and second reviewers (Y.K. and M.V.). Disagreements were resolved by discussion and third (E.B.) and fourth (B.W.) reviewers were consulted as necessary. This systematic review and meta-analysis was prospectively registered in the Open Science Framework (OSF). The OSF registration number was 9bc4y. Protocol details can be accessed via https://osf.io/9bc4y.

### Eligibility criteria

#### Type of participants

Studies that recruited adults aged 65 years or older were included. There was no restriction on the injury or disorder type, settings, and the history of falls. Animal studies and human studies with participants aged under 65 were excluded.

#### Type of studies

Studies conducted as a randomized control trial (RCT) and published in the English language were considered for inclusion. Studies with other research designs or non-peer-reviewed articles were excluded.

#### Intervention

Studies that employed all types of AE with a description of intervention details, such as duration, frequency, type, and intensity of AE, were included. The studies must have included a minimum of one AE group and a comparison group participating in another exercise program on dry land. Studies that did not include exercise components, such as bath or spa therapies, were excluded.

#### Outcome measures

Studies must have reported at least one outcome related to dynamic balance and compared the outcomes between AE and LE groups. All outcome measures must have been conducted on land because postural adjustment and movement patterns are significantly altered in water [21–23], and daily living activities are mostly performed on dry land. Studies including mixed intervention (e.g., both AE and LE in all groups) were excluded and any studies not providing data on the baseline or end-point outcomes were additionally excluded from the meta-analysis.

### Data extraction and coding

A total of 11 studies meeting the eligibility criteria were reviewed and coded in REDCap (https://www.project-redcap.org/). All relevant information was extracted for each study as follows: (1) report characteristics (2) participants (3) AE settings (4) interventions (5) outcome measures (6) results. The included studies were assessed and coded independently by two reviewers (Y.K. and M.V.) and discussed for consensus. If there was a disagreement, the study was re-evaluated to achieve consensus.

### Risk of bias and publication bias assessment

The analysis of the methodological quality and risk of bias of the included studies was conducted using the Cochrane risk of bias tool (RoB 2) [24] independently by two authors (Y.K. and M.V.). The tool can be utilized to assess the impact of each potential source of bias, at the “low”, “high”, and “somewhat concerns” risk level, respectively. The following criteria that potentially affect the risk of bias were addressed: randomization process, deviation from intended interventions, missing outcome data, measurement of outcome, selection of the reported result, and overall bias. Any disagreements were discussed until consensus was reached and additionally arbitrated by the third (E.B.) and fourth (B.W.) reviewers if needed. “Small study effects” is a generic term for the phenomenon that smaller studies sometimes show different, often larger, treatment effects than large studies [25]. In meta-analysis, small study effects are a well-known challenging and critical issue that may threaten the validity of the study results, and the most well-known reason of the small study effects is publication bias [25]. The publication bias can be displayed graphically in funnel plots, thus, a small study effect was examined and interpreted through a test for funnel plot asymmetry [26]. In the absence of publication bias, the plot should be shaped like a symmetrical funnel with small studies scattered widely at the bottom of the graph and larger studies spread narrowly [25].

### Meta-analysis

The purpose of the meta-analyses was to compare the pooled effect size between the AE group and LE group on dynamic balance in older adults. For the post-intervention sample size, when all subjects at the baseline were followed up, assessed, and analyzed regardless of their compliance to the intervention (intention-to-treat), the data including means and standard deviations for each outcome measure were used on the preferential basis [27]. Otherwise, the data of subjects who completed a pre-determined intervention(s) and have measurable data at the primary end point without any major protocol violations (per protocol) were used [27]. When data were not reported in the article as means and standard deviations, we contacted the corresponding authors and requested the data.

Outcome measurements included in the meta-analysis were assigned into three categories: (a) dynamic steady-state balance (e.g., 5-m walk test, 10-m walk test, backward tandem walk), (b) proactive balance (e.g., FRT; Functional Reach Test, TUG; Timed Up and Go test, 8-ft up-and-go test), and (c) balance test batteries (e.g., BBS; Balance Berg Scale and BOOMER; Balance Outcome Measure for Elder Rehabilitation) [28]. Where a trial reported more than one outcome in one of these categories, only one outcome with the highest priority was used for the analysis in line with Lesinski et al. [29]. The highest priority was given to the gait speed in the dynamic steady-state balance, FRT in the proactive balance, and BBS in the balance test battery [29]. When these representative outcomes were not available, the most similar outcomes related to the temporal (duration) and spatial (form of the motion) structure were used [29]. For a crossover RCT study [30], first-phase data were used. Sensitivity analyses were additionally performed to explore the robustness of the results by quantifying the differences in outcomes when removing one trial with a distinctly different direction of change in each category of balance outcome measurements.

The effect sizes between AE and LE groups were described as standardized mean differences (SMD) and 95% confidence intervals (CI). An effect size (SMD) 0.2–0.5, 0.5–0.8, and > 0.8 were considered a small, moderate, and large effect, respectively [31]. In case of a lower score indicating better performance in dynamic balance, scale directions were adjusted by multiplying − 1 to data, which resulted in a positive value indicating an improvement in favor of AE. For all analyses, we used an inverse-variance weighted random-effects model. All meta-analyses were performed using the Cochrane Collaboration’s Review Manager Software (RevMan 5.3.).

## Results

### Study selection

**Fig. 1:**
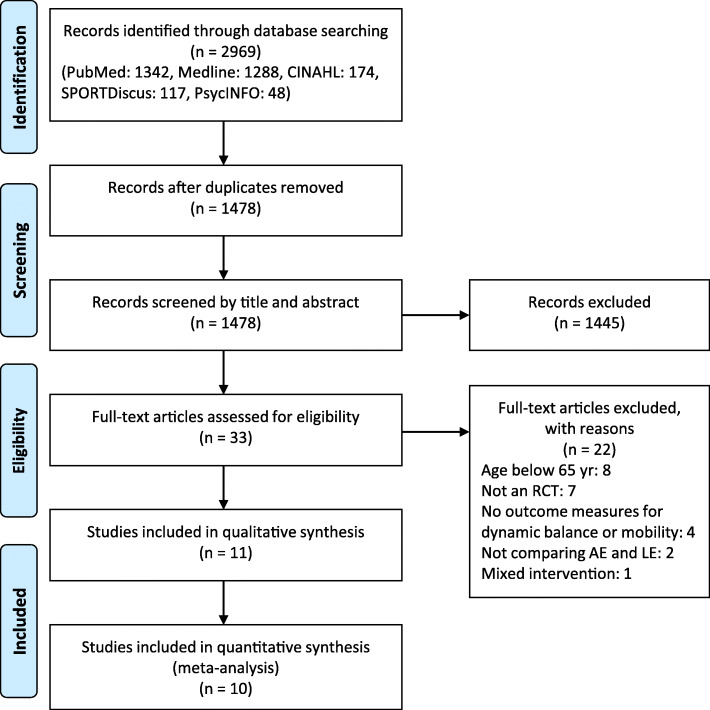
PRISMA flow diagram of article selection process

The electronic search retrieved a total of 2969 potential studies in the five databases, and no additional studies were identified by hand searching. Of these studies, 1491 duplicates were removed, and 1445 studies were excluded based on title and abstract content. We obtained the full text of the remaining 33 trials, 22 of which were excluded because they did not meet eligibility criteria. Finally, 11 studies were retained for our systematic review, and 10 studies were included in the meta-analysis after excluding one study due to insufficient data [32]. The flow diagram in Fig. 1 schematizes the steps of the selection of the studies.

### Characteristics of included studies

#### Participants

Eleven studies included in this systematic review were randomized controlled trials, which compared the impacts of AE and LE on dynamic balance in older adults aged 65 years or older. Table 1 presents the characteristics of participants of the 11 eligible studies that provided data for 372 participants with the mean age of 69.6 ± 4.0 years. The participants were recruited from community [11, 32, 33], hospital [30, 34, 35], and Parkinson’s associations [36–38]. Attrition rates were calculated using the following formula: Number of participants lost at post-intervention/number of participants at baseline*100. The attrition rates ranged from 0 to 27%.

**Table 1:** Characteristics of participants and exercise environments

Study	Group	Sample size (post-intervention)	Drop-outs (attrition rate: %)	Age: mean (SD)	Diagnosis	Type of pool/Gym	Water depth	Water/Room temperature (°C)
Adsett et al. 2017 [30]	AE	36 (33)	3 (8%)	72.9 (8.4)	Heart failure	Heated pool in hospital	Chest level	33–34
	LE	25 (25)	0 (0%)	68.3 (11.3)		Gymnasium in the hospital	NA	NR
Arnold et al. 2008 [11]	AE	21 (16)	5 (24%)	68.6 (5.4)	Osteoporosis	Community pool	Varied from shoulder to waist	30
	LE	20 (15)	5 (25%)	69.1 (6.3)		Community gym	NA	NR
Avelar et al. 2010 [32]	AE	14 (12)	2 (14%)	68.0 (5.7)	Healthy	Physical therapy pool	NR	NR
	LE	15 (14)	1 (7%)	69.0 (5.6)		Physical therapy gym	NA	NR
Bergamin et al. 2013 [34]	AE	20 (17)	3 (15%)	Total: 71.2 (5.4)	Healthy	Hot spring water	1.3–1.8 m	36.2
	LE	20 (17)	3 (15%)			NR	NA	20.1
Pérez de la Cruz et al. 2017 [37]	AE	15 (15)	0 (0%)	66.8 (5.3)	Parkinson’s	Indoor pool	1.1–1.45 m	30 (room: 27.5)
	LE	15 (15)	0 (0%)	67.5 (9.9)		Gym (varied)	NA	NR
Pérez de la Cruz et al. 2018 [36]	AE	14 (14)	0 (0%)	65.9 (7.1)	Parkinson’s	Indoor pool	1.1 m	30 (room: 27.5)
	LE	15 (15)	0 (0%)	66.4 (5.7)		NR	NA	NR
Simmons and Hansen 1996 [33]	AE	13 (10)	3 (23%)	82.0 (5.4)	Healthy	Outdoor pool	1–1.4 m (between waist and nipple line)	29.4–32.2
	LE	13 (12)	1 (8%)	78.2 (5.8)		Carpeted indoor church hall	NA	NR
Vivas et al. 2011 [38]	AE	6 (5)	1 (17%)	65.7 (3.7)	Parkinson’s	City spa	1.3 m	32
	LE	6 (6)	0 (0%)	68.3 (6.9)		NR	NA	NR
Volpe et al 2014 [39]	AE	17 (17)	0 (0%)	68.0 (7.0)	Parkinson’s	NR	NR	NR
	LE	17 (17)	0 (0%)	66.0 (8.0)		NR	NA	NR
Volpe et al. 2017 [40]	AE	15 (13)	2 (13%)	70.6 (7.8)	Parkinson’s	Therapeutic swimming pool	Chest level (Mammillary line)	NR
	LE	15 (11)	4 (27%)	70.0 (7.8)		NR	NA	NR
Zivi et al., 2018 [35]	AE	21 (21)	0 (0%)	66.3 (13.0)	Peripheral neuropathies	Heated swimming pool	NR	32
	LE	19 (19)	0 (0%)	71.8 (7.7)		NR	NA	NR

*AE* aquatic exercise, *LE* land exercise, *NR* not reported, *NA* not available

#### Aquatic setting and interventions

First, focusing on the pool characteristics, 10 studies reported the type of pool where the AE took place: Five at indoor swimming pools, three at therapeutic pools, two at outdoor swimming pools, and one not reported. The water depth varied from 1 m to 1.8 m, and the water temperature ranged between 27.5 °C and 36.2 °C (31.5 ± 2.6 °C) with an exception of three studies not reporting the aquatic setting [32, 39, 40]. The characteristics of pools are reported in Table 1.

The AE programs exhibited substantial differences across all included studies in regards to the intervention duration (45–60 min), frequency (1–5 sessions per week), and total duration (4–20 weeks) (Table 2). The AE programs identified included gait, mobility, stretching, stabilization, resistance, balance, endurance, strengthening, aerobic training, and Ai Chi. The exercises provided for AE and LE groups had the same or similar types, volume, emphasis, and objectives, except for two studies [36, 37]. Table 2 presents a summary of the exercise programs.

**Table 2:** Summary of exercise program

Study	Administrator	Dosage		Total duration (week)		Warm-up (min)	Main exercise (min)	Cool down (min)	Exercise details	Individually adjusted intensity	Aids/equipment for AE
		Min/session	Time/week								
Adsett et al. 2017 [30]	Physical therapist	60	1	6		Yes (time NR)	45	Yes (time NR)	Upper and lower limb endurance and resistance exercises	Y (RPE)	Cycling, steps, hand paddles, floatation rings
Arnold et al. 2008 [11]	Physical therapis	50	3	20		15	30	5	Gait, postural correction, upper/lower extremity mobility and stretching, trunk stabilization, resistance exercises, balance	Y (RPE)	Music, paddleboards, small weights, flotation devices
Avelar et al. 2010 [32]	NR	NR	2	6		3.5	NR (reps: 4 × 20)	3	Endurance exercises	NR	NR
Bergamin et al. 2013 [34]	Exercise trainer	60	2	6		8	50	8	Lower and upper body exercises (joint mobility, strengthening)	Y (RPE)	Not used
Pérez de la Cruz et al. 2017 [37]	Physical therapist	45	2	10	AE	Yes (time NR)	35	Yes (time NR)	Aquatic Ai Chi	NR	NR
					LE	10	25	10	Strength and aerobic exercises		
Pérez de la Cruz et al. 2018 [36]	Physical therapist	45	2	11	AE	Yes (time NR)	30	Yes (time NR)	Aquatic Ai Chi	NR	NR
					LE	10	30–40	20	Strength and aerobic exercises		
Simmons and Hansen 1996 [33]	NR	45	2	5		NR	45	NR	Gait training	NR	NR
Vivas et al. 2011 [38]	Physical therapist	45	2	4		10	35	0	Trunk mobility, postural stability training, dynamic balance	Y	Flotation devices, water turbulence, balance plate, stick and hoop
Volpe et al. 2014 [39]	NR	60	5	8		10	40	10	Perturbation-based balance training	NR	NR
Volpe et al. 2017 [40]	Physical therapist	60	5	8		10	40	10	Exercises for postural deformities	NR	Flotation device
Zivi et al., 2018 [35]	Physical therapist	60	3	4		NR	60	NR	Balance, posture control, and gait exercises	NR	Treadmill, cycloergometer, cyclette, stabilometric platform

*AE* aquatic exercise, *LE* land exercise, *NR* not reported, *RPE* the Borg rating of perceived exertion scale

#### Outcome measurements and summary of the results

All studies included in this review performed at least one dynamic balance-related measurement before and after the intervention on land. Four studies evaluated long-term effects at additional stages after the intervention was terminated [36–38, 40], but the second post-intervention outcome measure data were not used due to differences in the time points after interventions and limited data. Overall, eight studies reported greater improvements in AE groups compared to LE groups in at least one dynamic balance outcome measurement [11, 33–39], whereas two studies did not find any statistically significant differences between AE and LE groups [32, 40], and one study reported a greater improvement in LE group in one outcome measurement [30]. Table 3 presents the details of outcome measurements and a brief summary of the results of individual studies.

**Table 3:** Outcome measures and summary of main findings of all selected studies

Study	Outcome measures	Follow-up	Adverse events	Participants feedback	Results
Adsett et al. 2017 [30]	6MWT, **TUG**, **10-m walk test (speed)**, **BOOMER**	N	Shortness of breath (1), dizziness (2)	Reported	LE group showed greater improvements in 6MWT. No significant differences in 10-m gait speed and BOOMER.
Arnold et al. 2008 [11]	**BBS**, **FRT**, **backward tandem walk**	N	Pain: 29% AE, 52% LE. Muscle cramping and stiffness: 25% AE, 3% LE	NR	AE group showed a greater improvement only in the backward tandem walk versus LE group. No significant differences in BBS and FRT between two groups.
Avelar et al. 2010 [32]	DGI, BBS, Tandem gait test, 10-m gait speed test	N	NR	NR	Both intervention groups showed improvements only in DGI and BBS, with no difference between groups.
Bergamin et al. 2013 [34]	**8-foot up-and-go test**	N	None	NR	Both intervention groups showed improvements, with significantly greater improvement in AE group.
Pérez de la Cruz et al. 2017 [37]	**BBS**, Tinetti Scale, FTSTS, **TUG**	1 month	None	NR	Only AE group showed improvements in all variables, except the FTSTS. LE group showed no improvements in any of the balance measures.
Pérez de la Cruz et al. 2018 [36]	**TUG**, FTSTS,	1 month	NR	NR	AE (Ai Chi) group showed improvements in TUG and FTSTS in post-treatment and 1-month follow-up, whereas the dryland group showed no significant differences.
Simmons and Hansen 1996 [33]	**FRT**	N (10–12: injury tracking)	NR	NR	AE group showed gradual improvements in each week. LE group showed improvement only in the initial week. At week 5 (post), AE group showed significant improvement compared to LE groups.
Vivas et al. 2011 [38]	**FRT**, **BBS**, **5-m walk test**, TUG	17 days	NR	NR	Both exercise groups showed improvements in FRT. Only the AE group improved in the BBS.
Volpe et al 2014 [39]	**Instrumental version of FRT**, TUG, **BBS**,	N	None	NR	Both groups showed improvements in all outcome variables, with a better improvement in AE group BBS.
Volpe et al. 2017 [40]	**TUG**, **BBS**	2 months	NR	NR	Both groups showed improvements in all parameters, with no intergroup differences.
Zivi et al., 2018 [35]	**BBS**, Dynamic Gait Index	N	NR	NR	AE group showed a greater improvement in the Dynamic Gait Index. No significant difference in BBS between groups.

Outcome measurements included in the meta-analysis were highlighted (bold), *AE* aquatic exercise, *LE* land exercise, *NR* not reported, *DGI* Dynamic gait index, *BBS* Berg Balance Scale, *FTSTS* Five Times Sit-to-Stand test, *TUG* Timed Up and Go test, *FRT* Functional Research Test, *6MWT* 6-min walk test, *BOOMER* Balance Outcome Measure for Elder Rehabilitation

#### Risk of bias and publication bias

**Fig. 2:**
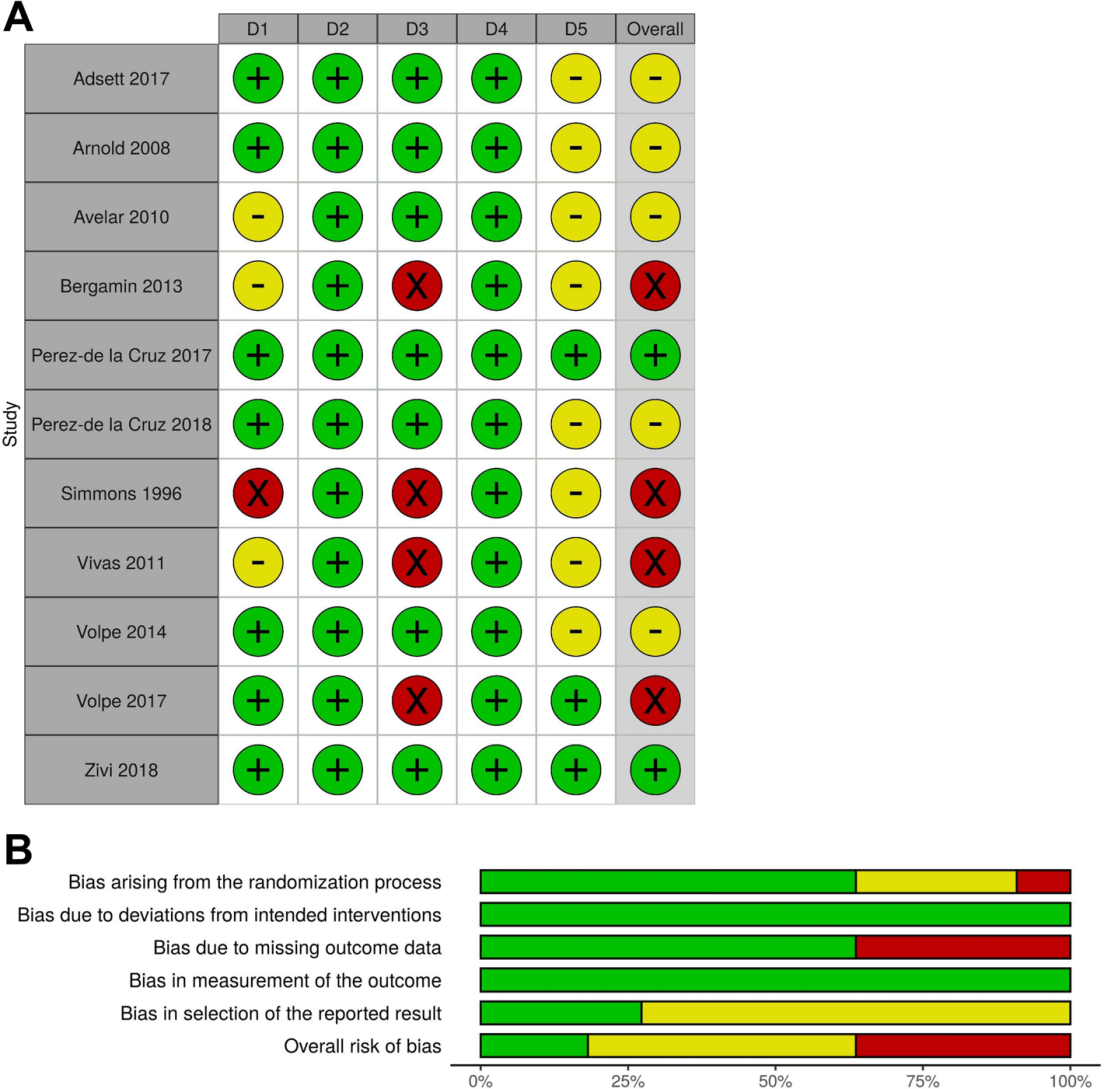
Risk of bias of the included studies. **a** Risk of bias graph, **b** Risk of bias summary. Green, low risk; yellow, somewhat concerns; red, high risk. D1, Randomization process; D2, Deviation from intended interventions; D3, missing outcome data; D4, measurement of outcome; D5, selection of the reported result; Overall, overall bias

**Fig. 3:**
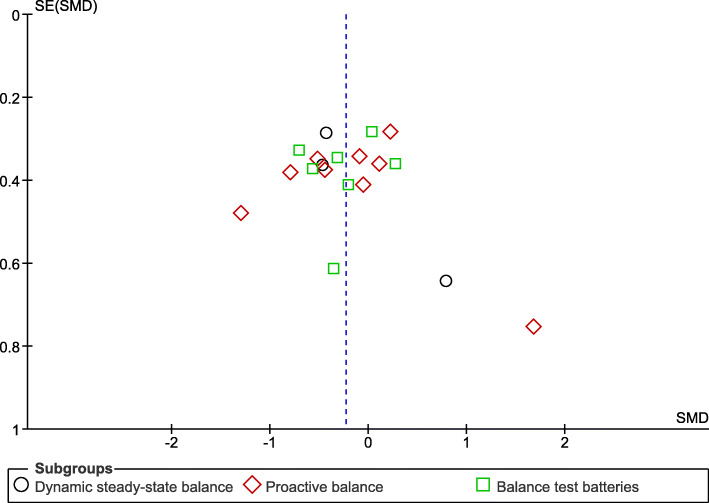
Funnel plot for all of the meta-analyses

The Cochrane risk of bias tool indicated a “low” risk of bias for two studies [35, 37] and “high” risk of bias for four studies [33, 34, 38, 40] due to randomization process [33] and missing outcome data [33, 34, 38, 40]. The other five studies had “somewhat concerns” [11, 30, 32, 36, 39] due to randomization process [32] and selection of the reported result [11, 30, 32, 36, 39]. Figure 2 presents the risk of bias of the included studies. The visual inspection of the funnel plot identified substantial asymmetry, indicating the possibility of publication bias in the meta-analysis (Fig. 3).

### Meta-analysis

Post-intervention assessment data for BBS, Dynamic Gait Index, tandem gait, and 10 m gait speed from the study by Avelar et al. [32], data for 5-m walk test, FRT, and TUG from the study by Vivas et al. [38], data for BBS from the study by Arnold et al. [11], and data for 10-m gait speed and BOOMER from the study by Adsett et al. [30] were requested, and all data, except those from the study by Avelar et al. were received. Thus, a total of 10 studies were included in the meta-analysis of dynamic balance outcomes for AE compared with LE [11, 30, 33–40].

**Fig. 4:**
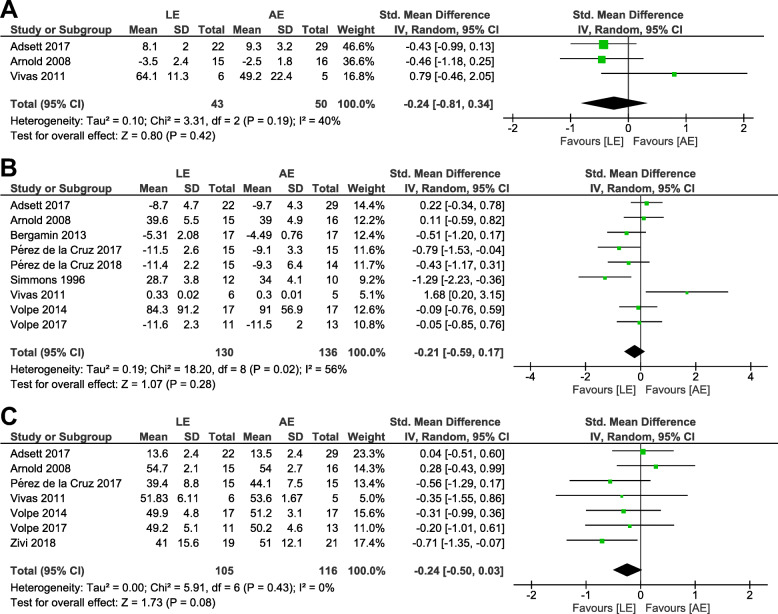
Forest plot of comparison: AE versus LE. **a** Dynamic steady-state balance, **b** Proactive balance, **c** Balance test batteries

**Fig. 5:**
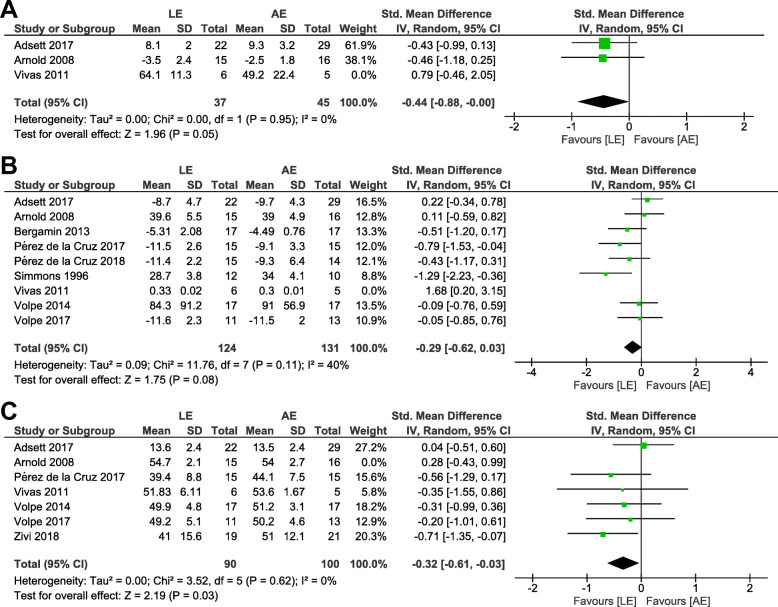
Results of sensitivity analyses. **a** Dynamic steady-state balance, **b** Proactive balance, **c** Balance test batteries

Outcome measurements included in each category were as follows: (a) dynamic steady-state balance: 10-m walk test (speed) [30], 5-m walk test (speed) [38], and backward tandem walk (number of errors) [11], (b) proactive balance: FRT [11, 33, 38, 39], TUG [30, 36, 37, 40], and 8-ft up-and-go test [34], (c) balance test batteries: BBS [11, 35, 37–40] and BOOMER [30]. When a random-effect analysis was applied using the 10 studies involving 343 participants, AE groups compared with LE groups displayed comparable improvements in dynamic steady-state balance (SMD = − 0.24; 95% CI, −.81 to .34), proactive balance (SMD = − 0.21; 95% CI, −.59 to .17), and balance test batteries (SMD = − 0.24; 95% CI, −.50 to .03) (Fig. 4). The sensitivity analyses after excluding one trial with a distinctly opposite direction of change in each category presented that the point estimates changed by − 0.20 (SMD = − 0.44; 95% CI, −.88 to 0) in dynamic steady-state balance, by − 0.08 (SMD = − 0.29; 95% CI, − 62 to .03) in proactive balance, and by − 0.08 (SMD = − 0.32; 95% CI, −.61 to −.03) in balance test batteries (Fig. 5).

## Discussion

This is the first systematic review with meta-analysis comparing the effects of AE and LE on dynamic balance in older adults. Eight of the included studies [11, 33–39] concluded that AE resulted in greater improvements in at least one dynamic balance outcome measurement compared to LE. However, the results of the meta-analysis revealed no statistically significant differences in all outcome categories. This result is consistent with a previous review conducted by Waller et al. that compared the effects of aquatic and land-based exercise programs on physical functioning in healthy older adults and demonstrated small effect sizes in postural stability in favor of AE and in walking ability in favor of LE [13]. In consideration of the limited number of studies included in this analysis and results of the sensitivity analyses, however, the results must be interpreted with caution.

Although different musculoskeletal or neurological disorders do not share identical signs or symptoms, dynamic balance is important across all older populations to prevent fall risk and to enhance rehabilitation from fall-related injuries. For example, Parkinson’s disease is a degenerative neurological disorder commonly reported in the senior population, and the risk of falls and fall-related injuries increase in this population due to deficits in motor functions and postural stability [41]. Osteoporosis, which is also common in the senior population, reduces the bone density and results in a higher risk of fractures caused by falling [42]. In addition, those with osteoporosis commonly show muscle weakness, postural deformity, and deteriorated postural control that may significantly increase the risk of falls and fractures [43, 44]. Thus, various balance abilities have to be trained from both preventive and rehabilitative perspective in those populations. Moreover, dynamic balance is a common interest in all senior populations regardless of the disorder because aging brings a natural biological degeneration in regards to muscle strength and mass and neurological functions [45]. Thus, older adults without any disorder also present a greater risk of falls when compared to younger adults due to inappropriate muscular activation and control of the body’s center of mass during ambulation (e.g., dynamic balance) [46]. The comparably effective AE and LE in overall older adults suggests that participants can select the training environment based on their preference.

### Intervention and outcomes

Postural strategies vary in different environments regardless of age and physical fitness [16]. Both older and younger adult populations demonstrated the greatest postural sway and sway velocity with the lowest perceived stability in chest-deep water compared to the same measures made at shallow water depths and on land [16, 22, 47]. However, none of the trials included in this current review provided a rationale for the water depth chosen and considered each participant’s height. Although all studies recruited both male and female participants with different mean height, except for only one trial by Arnold et al. [11], the AEs were conducted in water with the non-adjustable water level. That implies the participants in the AE groups were trained with all different exercise intensities despite the identical location, settings, and exercise types. In addition, movement patterns and mechanical power outputs during the same physical performance are presented differently in water and on land [48]. Thus, although most of the trials included provided the same or similar exercise programs to both AE and LE groups, the subjective exercise intensities can be different due to the environmental factors, which may affect the ultimate training effects. The main reason AE is recommended to the older adults is to utilize the physical properties of water and provide an optimized medium for exercise. Therefore, it is recommended that future studies provide rationales for water depth and exercise intensities in all intervention groups to investigate and compare the effects between AE and LE more accurately.

The intervention dose, duration, intensity, and type of exercise varied considerably in each trial, but there was no justification for the exercise dose chosen. According to ‘The 2018 Department of Health and Human Services’ guideline [49], older adults should get at least 150 min per week of moderate-intensity or 75 min per week of vigorous-intensity aerobic activity with moderate or high-intensity muscle-strengthening activities at least 2 days a week. Specifically, it is recommended for older adults with the risk of falls to participate in balance training three or more times per week to reduce falls. Older adults in three trials participated in AE and LE at least 150 min per week [11, 39, 40], and those in two trials practiced balance training at least 3 times per week [11, 39]. The intensity of the activities can be perceived in different ways according to various factors, such as physical fitness, muscular performance, or level of disorder or degeneration. Only two studies [11, 34] assessed subjective exercise intensity using the Borg rating of perceived exertion scale (RPE scale), and participants were instructed to exercise at a predetermined intensity. However, the optimal dosage, duration, and intensity of AE were not identified as most of the studies demonstrated low-to-moderate effect sizes and both AE and LE groups mostly presented comparable results across all trials.

The outcomes were measured using various dynamic balance tests, but the assessments were performed immediately after the interventions were terminated. Although each measurement contains critical components in daily living activities and indirectly predicts the potential risk of falls, the generalization of the results regarding the reduction of fall risks must be interpreted with caution as these are lacking in regards to the longer effects of the interventions. Therefore, future studies may wish to evaluate dynamic balance in an extended length of time to assess endurance-related muscle functions that are also essential for postural adjustment in daily life. The aim of AE interventions in the older population is to improve physical fitness, functional performance, and postural adjustment to ultimately reduce the risk of falls and fall-related injuries and improve their quality of life. Simmons and Hansen [33] tracked the rate of injuries between 10 and 12 months after the termination of the last session and reported that there were no orthopedic injuries from falls in the AE group, whereas there were two bone fractures (16.7%) in the LE group since the last session. Two trials conducted by Pérez de la Cruz et al. [36, 37] also included second post-intervention assessments, but the time interval (1-month post-intervention) was not sufficient to determine long term effects of AE on dynamic balance or fall reductions. Arnold et al. [11] and Volpe et al. [39] reported adverse events that occurred during the interventions, but none of the included studies reported participants’ feedback for the AE or LE programs. Besides the main outcome measures, supplementary information regarding injuries and psychological effects, such as satisfaction and enjoyment, may be helpful for an in-depth interpretation of the effectiveness of AE.

In consideration of the exercise program components, the results of the meta-analyses that demonstrated AE and LE have equivalent effects on dynamic balance should be interpreted with caution. In general, to improve a specific skill, a completely or nearly identical task is generally included in exercise interventions to induce a practice effect. However, among the ten trials in the meta-analyses, only four trials included at least one balance or gait-related task in the exercise programs [11, 35, 38, 39], and the rest of the ten trials included other types of exercises, such as endurance, strength, mobility, or aerobic exercises, that may contribute to the improvement of dynamic balance. Thus, future research may wish to include a goal-focused exercise program that focuses on balance-related tasks and controls for other variables, such as exercise intensity, to more clearly compare the effectiveness of AE and LE on dynamic balance in the older population.

### Clinical implication

This study did not identify the statistical superiority of AE over LE programs on dynamic balance. However, these results imply that AE can be an appropriate alternative to LE which leads to clinically meaningful improvements in balance. Both AE and LE have different advantages. Because LE is performed under dryland conditions and is more associated with activities of daily living, these can be more applicable and transferable to enable older adults to successfully improve practical skills. Due to environmental characteristics, muscle activation patterns and movement kinematics are different during aquatic activities compared to those during identical land activities [23, 50], which may lead to less transferability to various functional tasks on dry land, however, this has not been formally tested or observed in previous research. The aquatic environment provides older adults with numerous biological, neurological, and musculoskeletal advantages and helps them perform higher exercise intensities in a safer and supportive training environment without the risk or fear of falling [14, 48, 51–54]. Therefore, it is suggested that future studies and practitioners select the proper exercise mode that matches each participant’s preference and aim of the intervention to maximize the intervention effectiveness. Further investigations regarding the classification of disorder, disease, or history of falls may provide stronger scientific rationales for future balance training protocols for older adults.

As identified in this review, most of the AE programs were administered by physical therapists in clinical facilities. Because of the limited accessibility of aquatic exercise facilities, availability of experts, and higher medical costs, AEs are not broadly practiced in the senior populations. Thus, more easily accessible and lower-cost AE protocols need to be established so that older adults can participate in various physical activities in a safer environment to improve balance, reduce the risk of falls, and ultimately improve their quality of life.

### Study limitations

This systematic review and meta-analysis have several limitations. First, this study was limited to peer-reviewed journal articles published in English and RCT designs only, which may increase the risk of publication bias and potentially exclude appropriate studies with high-quality methodologies. In consideration of the potential small study effects and publication bias, future meta-analyses may want to identify and include unpublished outcomes and unpublished studies to improve the validity of results [55]. Also, we included outcomes using the balance categories instead of using just one measure from each study because we only had 10 studies. Due to the small number of studies included in each category, potential covariates, such as the duration of intervention, exercise type, or exercise intensity, could not be appraised using a moderator analysis. In future reviews, it may be appropriate to use a single measure in each study and conduct a meta-regression to identify the impacts of the potential covariates on the effect sizes in the meta-analyses. In addition, five out of 11 studies in the review presented “somewhat concerns” of risk of bias and four had a “high” risk bias, that potentially cause overestimation of the true effects of AE and LE. The randomization process, missing outcome data, and selection of the reported result were the main causes of bias. Thus, we suggest that future trials make advanced plans for these three categories. Furthermore, as only two outcomes [33, 38] in the proactive balance category demonstrated high effect sizes, we were not able to establish the general guideline with optimal exercise type, intensity, dosage, and duration to improve dynamic balance in older adults.

## Conclusion

To summarize, AE displays comparable effects on dynamic balance in older adults aged 65 years or older when compared to LE. Thus, AE may be effectively utilized as a safer alternative to LE, but the results should be interpreted with caution due to the limited quantity and risk of bias of the studies. Considering clinical applications, further trials with longer-term outcome measures are needed to elucidate effective AE protocols on balance and falls.

## Data Availability

The datasets generated and analyzed during the current study are available from the corresponding author on reasonable request.

## Abbreviations

PRISMA: Preferred reporting items for systematic reviews and meta-analysis
AE: Aquatic exercise
LE: Land exercise
